# A Rare Case of Primary Purulent Pericarditis Caused by *Streptococcus constellatus*

**DOI:** 10.3390/medicina59010159

**Published:** 2023-01-13

**Authors:** Medeinė Kapačinskaitė, Dovilė Gabartaitė, Agnė Šatrauskienė, Ieva Sakaitė, Vytė Valerija Maneikienė, Aleksejus Zorinas, Vilius Janušauskas

**Affiliations:** 1 Faculty of Medicine, Vilnius University, M.K. Čiurlionio g. 21, 03101 Vilnius, Lithuania; 2 Department of Cardiology, Center of Cardiology and Angiology, Vilnius University Hospital Santaros Clinics, Santariškių g. 2, 08661 Vilnius, Lithuania

**Keywords:** pericarditis, *Streptococcus constellatus*, pericardiocentesis, cardiac tamponade, pericardiectomy

## Abstract

*Background*: Bacteria-caused acute pericarditis is a very rare entity. It is usually associated with an underlying infection or compromised immune system. Primary purulent pericarditis in a previously healthy individual is highly unexpected; therefore, it is likely to have a delayed diagnosis and poor outcomes. *Case*: We report a case of an adult immunocompetent patient with primary bacterial pericarditis caused by a member of the commensal oral flora *Streptococcus constellatus.* The patient presented with septic shock and cardiac tamponade, and was further complicated with constrictive pericarditis, which was successfully treated with pericardiectomy. *Conclusions*: Bacterial pericarditis is a fulminant disease with a high mortality and complication rate. Fast recognition and prompt therapy are required to achieve a full recovery.

## 1. Introduction

Acute pericarditis is a common pericardial disease and a relatively common cause of hospital admissions due to chest pain [1]. Clinical manifestation and severity of the disease can differ greatly in every case. Bacterial pericarditis tends to have a more fulminant course, and a higher complication and mortality rate [2,3]. The use of antibiotics has made bacterial pericarditis extremely rare and nowadays, it is mostly presented in immunocompromised patients or as a secondary infection caused by hematogenous spread from the initial source of infection [4]. Here, we present a case of a 45 year old immunocompetent previously healthy patient who was diagnosed with primary purulent pericarditis caused by *Streptococcus constellatus*.

## 2. Case Presentation

**Figure 1:**
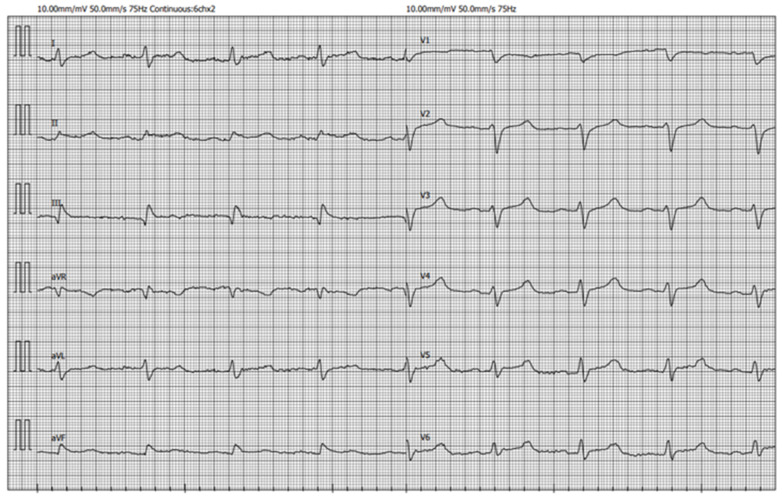
Admission ECG showing sinus tachycardia with slightly elevated ST segment in II, aVF leads.

A 45 year old male was admitted to the emergency department with progressive shortness of breath. The patient stated that he had a fever of up to 37.5 °C over the previous few days. Additionally, he denied any chronic diseases and allergies. At the time of presentation, the patient was well-oriented but hemodynamically unstable: he had tachycardia with a heart rate of 105/min, his blood pressure was 50/30 mmHg, and his oxygen saturation was 60%. Auscultation showed decreased vesicular breath sounds and normal heart sounds without murmurs or friction rub. Examination of the abdomen and extremities was unremarkable except for prolonged capillary refill time. Complete blood count showed leukocytosis of 39.05 × 10^9^/L with left shift. Other significant blood tests were as follows: C-reactive protein (CRP) 299 mg/L, procalcitonin 8.82 μg/L, serum creatinine 224 μmol/L, aspartate aminotransferase 3672 IU/L, alanine aminotransferase 1540 IU/L, potassium 6,7 mmol/L, sodium 120 mmol/L, d-dimer 22,150 μg/L, brain natriuretic peptide 656 ng/L. Arterial blood gas analysis showed acidosis and respiratory failure with pH 7.015, pO_2_ 26.3 mmHg, pCO_2_ 33.6 mmHg, and HCO_3_ 8.2 mmol/L. Initial ECG demonstrated sinus tachycardia with a slight ST segment elevation in II and aVF leads (Figure 1).

**Figure 2:**
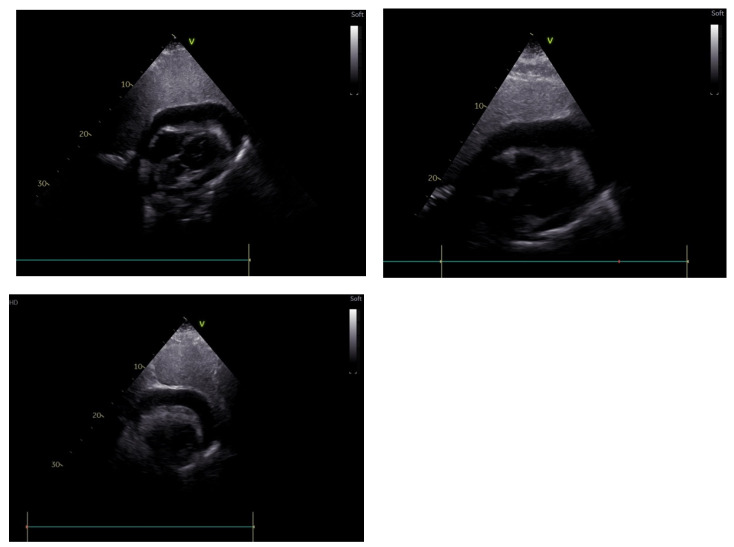
Transthoracic echocardiogram showing a large pericardial effusion (most prominent around the left ventricular posterior wall and the left ventricular apex with about 2.7 cm and 2.4 cm in diameter, respectively) with a ventricular systolic collapse and a ‘’swinging heart’’ view.

**Figure 3:**
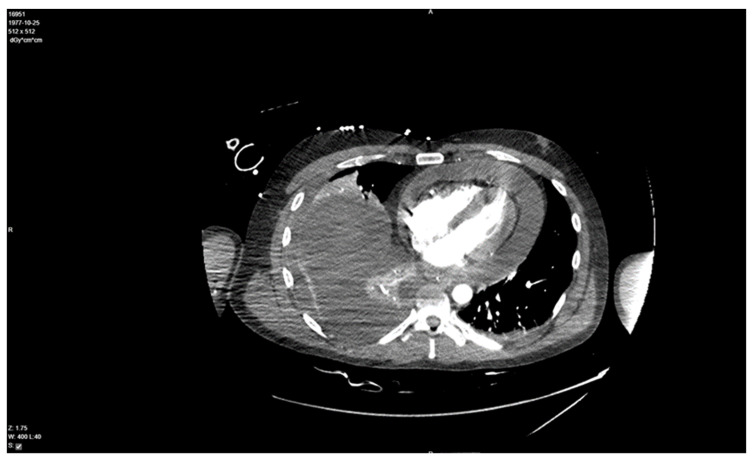
Chest CT demonstrates right-side effusion and a 26 mm thickness buildup of fluid in the pericardial space.

**Figure 4:**
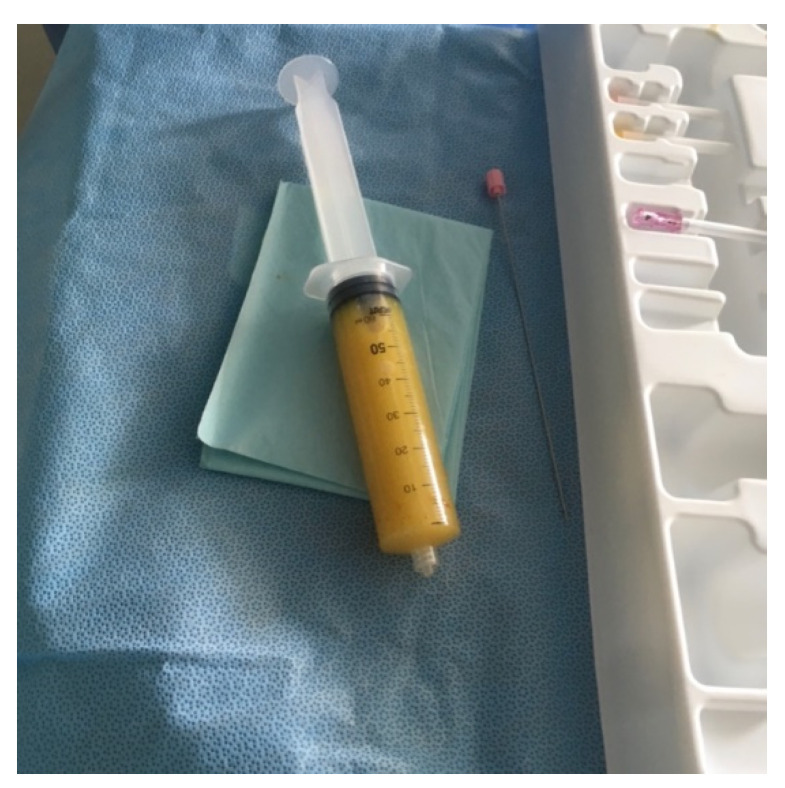
Pericardial punctate with thick yellowish pus.

In the screening echocardiography, large pericardial effusion with signs of compression was observed, raising suspicion of cardiac tamponade (Figure 2). In addition to echocardiographic findings, the chest CT showed right-sided pleural effusion (Figure 3). A diagnosis of septic shock with multiple organ dysfunction and hypoxemic respiratory failure was made and the patient was admitted to the intensive care unit. Empiric antibiotic treatment with meropenem and vancomycin was initiated. Abdominal and pelvic CT did not show any abnormalities. Alongside the treatment for septic shock and respiratory failure, pericardiocentesis was performed and a pericardial drainage catheter was placed. About 700 mL of thick purulent fluid was drained over two days (Figure 4).

The patient’s overall condition improved significantly, and on the third day, he was transferred to the cardiology department. *Streptococcus constellatus* was isolated from the pericardial fluid and the antibiogram showed susceptibility to ampicillin. Antibacterial therapy was changed accordingly to intravenous ampicillin 2 g every 6 h. A diagnosis of acute purulent pericarditis was made. In addition to the antibiotics, oral ibuprofen (600 mg three times per day) and colchicine (0.5 mg twice per day) were added to the treatment plan. Therapy with ampicillin, colchicine, and ibuprofen continued for one month and ceased after the pericardiectomy was performed. An oral and dental examination showed no signs of primary infection source. The patient also tested negative for HIV, HBV, and HCV, and a further search for possible immunodeficiency was postponed until normalization of the inflammatory process. The urine test was also negative. No more pericardial effusion was evacuated and the drain was removed after 1 week. There was a significant improvement in the patient’s condition and laboratory tests (CRP decreased 299 ≥ 30.2 mg/L, renal and liver function normalized).

**Figure 5:**
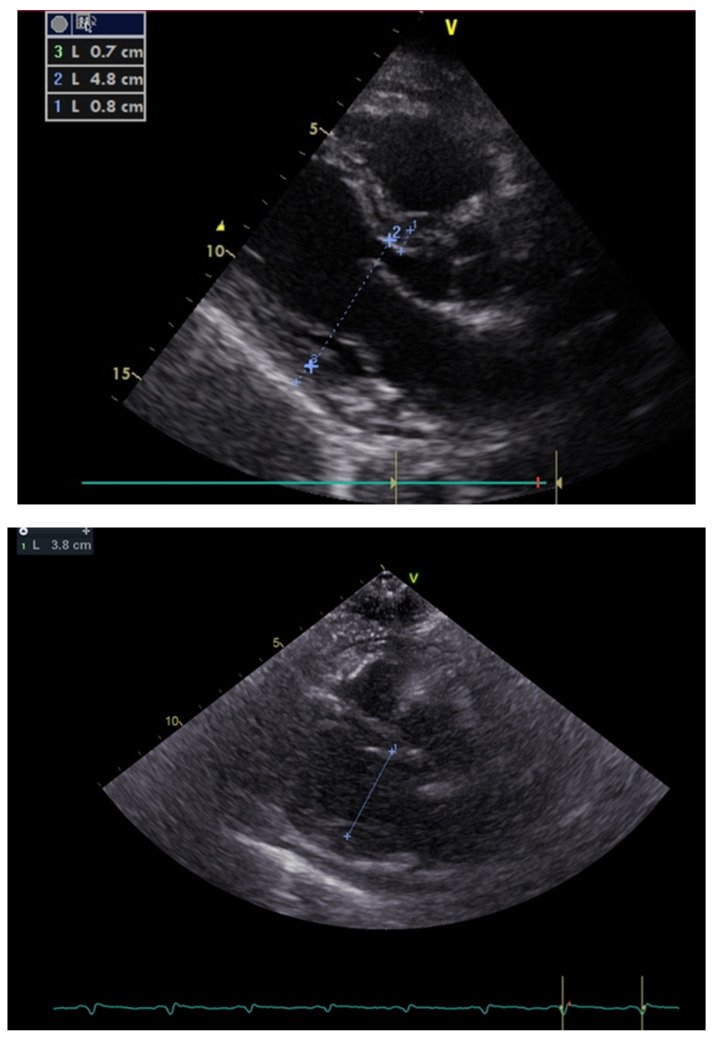
Markedly decreased left ventricular diastolic diameter from long axis parasternal view within 20 days.

At the end of the second week, the patient was diagnosed with *Cl. difficile* enterocolitis and was treated with oral metronidazole (500 mg every eight hours) and vancomycin (250 mg every six hours) for 12 days. Subsequently, he developed dyspnea, ascites, peripheral edema, and hypotension. Echocardiography revealed pericardial constriction, compression of both ventricles, a respiration-related intraventricular septal shift towards the left ventricle, marked respiratory variation in mitral and tricuspid inflow velocities (mitral Doppler velocity with an inspiratory decrease of 27% and tricuspid Doppler velocity with an increase of 33%), and an increase in the hepatic vein expiratory diastolic reversal flow (Figure 5).

**Figure 6:**
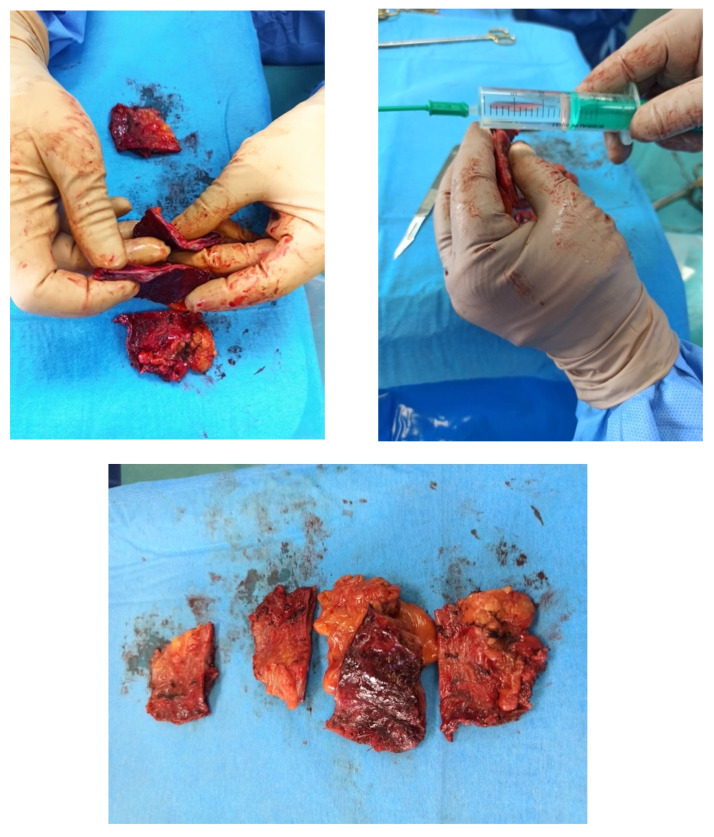
Gross pathological specimen of markedly thickened pericardium after pericardiectomy.

Due to progression of heart failure, a pericardiectomy was scheduled. In order to achieve better postoperative outcomes, it was decided to carefully monitor the patient’s condition and, if possible, to wait for the recovery from *Cl. difficile* enterocolitis. After two days, the molecular *Cl. difficile* test was negative. Due to the progression of symptoms and ineffective drug therapy, a decision to perform a pericardiectomy was made. Before the surgery, coronary angiography was performed to evaluate risks and postoperative outcomes. It showed 70% stenosis of the right coronary artery, the patient discussed with the heart team, and it was recommended to refrain from revascularization. The patient underwent pericardiectomy one week after the onset of dyspnea and one month after the admission to the hospital. However, due to complicated access to the posterior heart surface, a subtotal pericardiectomy was performed. Repeated echocardiography showed reduced constriction with residual adhesions to the posterior heart wall. The postoperative period was complicated by cardiogenic–septic shock with multiple organ failure and the patient was intubated. Inflammatory markers were significantly elevated: CRP 231 mg/L, procalcitonin 2.13 μg/L. In addition, a chest X-ray revealed bilateral pulmonary infiltrates, and postoperative ampicillin/sulbactam was empirically changed to piperacillin/tazobactam (18 g per 24 h). Blood cultures were negative, however, bronchial aspirate showed growth of *Klebsiella aerogenes* and *Serratia marcescens,* which were both susceptible to piperacillin/tazobactam. The bronchial aspirate culture was also positive for *Candida albicans*, therefore, fluconazole was added. Intra-aortic balloon counter-pulsation was applied to ensure sufficient cardiac output. Despite the treatment, the patient’s condition was deteriorating and hemodynamic instability persisted, therefore, a decision to perform a total pericardiectomy was made. The second surgery was carried out 1 week after the first (Figure 6).

After the surgery, *Klebsiella pneumoniae* was isolated from the tracheal aspirate and piperacillin/tazobactam was switched to meropenem. Gradually, the patient became hemodynamically stable and recovered from delirium, his inflammatory markers improved (CRP has decreased 231 ≥ 15.3 mg/L), and echocardiography demonstrated a full resolution of constriction. Subsequently, after almost two months in the hospital, he was discharged and referred to rehabilitation. During the follow-up visit two months after the second pericardiectomy, the patient was free of symptoms, veloergometry showed satisfactory exercise tolerance, and the echocardiogram was without evidence of constrictive pericarditis. Also, the patient was consulted by an infectious disease doctor, and immunodeficiency was not found. For outpatient treatment, 20 mg of torasemide, 25 mg of spironolactone, 20 mg of betaxolol, 100 mg of acetylsalicylic acid, and 80 mg of atorvastatin were prescribed to use once daily. The patient was signed up for an outpatient cardiology consultation in six months.

## 3. Discussion

Infectious pericarditis can be categorized by its causative agents into viral, bacterial, mycobacterial, fungal, and parasitic. Viruses are thought to be the leading cause of acute pericarditis, presumably responsible for the majority of idiopathic cases [4]. In confirmed viral pericarditis cases, echovirus, coxsackieviruses A and B, cytomegalovirus, and enterovirus are found most frequently [5,6]. The incidence of tuberculous pericarditis is low, nevertheless, it is still significant in developing countries and immunocompromised patients [4,6]. Bacterial pericarditis is a rare entity, causing less than one percent of all cases [2,7]. The most isolated are *Staphylococcus aureus* and *Streptococcus pneumoniae* [5,6].

In our presented case, purulent pericarditis was caused by *Streptococcus constellatus*. Together with *Streptococcus anginosus* and *Streptococcus intermedius*, it forms the Streptococcus anginosus group (SAG). These Gram-positive pathogens can normally colonize the upper respiratory, digestive, and genitourinary tracts. However, in favorable conditions, SAG can cause invasive or non-invasive pyogenic infections and involve multiple organs [8]. Since purulent pericarditis is mostly caused by hematogenous seeding of bacteria, it is important to find a primary infection source. Though SAG can cause infection in any part of the body, more commonly they are isolated from skin and soft tissue lesions, peritonsillar, appendiceal, liver, brain, and lung abscesses [8,9]. From a search of the PubMed database, we identified 13 reports of purulent pericarditis caused by SAG [10,11,12,13,14,15,16,17,18,19,20,21,22]. Out of these, only two cases were caused by *Streptococcus constellatus* [16,17]. In addition, five cases were described as primary pericardial inflammation, while others were secondary to pulmonary infection, liver abscess, gastropericardial fistula, cardiac surgery, advanced cancer, or as a complication of transbronchial needle aspiration. It is worth mentioning that as a part of oral microflora, SAG can spread to blood in patients with periodontal diseases [23]. Dental plaques and gingival bleeding after brushing teeth are also associated with an increased risk of bacteremia [24]. Another possible entry gate to the bloodstream is increased intestinal permeability. Similar to other pathogens, SAG can cross the damaged gastrointestinal barrier and cause infection in extraintestinal organs [8]. The work of Siegman-Igra et al. also pointed out possible SAG growth in urine in patients with urinary tract infections (UTIs) and clinically non-significant bacteriuria [25]. The majority of studies agree on predisposing factors for SAG infections. The significant risk factors for bacteremia are unhealthy diet, alcohol, smoking, dental and gastrointestinal surgery, underlying diseases such as diabetes, digestive tract malignancies, and chronic renal failure [8,9,25,26]. Apart from this, a significantly higher mortality rate was observed in patients over 65 years [26].

Clinical manifestations of purulent pericarditis can vary from subtle symptoms to rapidly deteriorating conditions. The defining features of acute pericarditis, such as retrosternal pleuritic chest pain, pericardial friction rub, and diffuse ST-segment elevation in ECG, can be absent or overwhelmed by non-specific symptoms of concomitant infection [17,20,27]. Fever can be an important differential feature, since it is less common in viral and idiopathic pericarditis [28]. Patients also frequently complain of dyspnea, which is associated with pericardial effusion and constriction [4]. It is essential to note that dangerous complications such as cardiac tamponade are more common in bacterial pericarditis and can be the first manifestation of a disease or occur in a short interval after initial symptoms [2,4,6]. Most cases of acute pericarditis do not require full diagnostic and etiologic evaluation, since they follow a benign course, however, large effusion, suspected cardiac tamponade, or purulent pericarditis have to be thoroughly examined [7,29]. High levels of inflammation markers, leukocytosis, and pathological changes in echocardiogram are important steps in diagnosis [29]. Complicated pericardial effusion with high echogenicity and septations in the pericardial space, together with clinical signs of sepsis, strongly indicate a bacterial cause [4].

If purulent pericarditis is suspected, immediate administration of intravenous broad-spectrum antibiotics should be initiated and later changed to culture-targeted therapy [4,29]. Aspirin or non-steroidal anti-inflammatory drugs, together with colchicine, are also used in treating pericardial inflammation [29]. Pericardiocentesis is indicated in patients with symptomatic or large pericardial effusion, cardiac tamponade, or possibly purulent pericarditis [30]. Drained pericardial fluid should be sent for cytology, bacterial, fungal, and mycobacterial cultures [6]. In case of a rapidly worsening condition, resistance to medical treatment, and complicated residual effusion, surgical pericardial drainage with saline irrigation should be considered [4]. Some studies reported that intrapericardial fibrinolysis was successful in preventing constriction and could be used to facilitate pericardial drainage of dense purulent effusions [30,31]. Complications and mortality rates in bacterial pericarditis are high [3,32]. The most common complications are cardiac tamponade and transformation to pericardial constrictions [4,27]. In 13 reviewed cases of SAG-caused pericarditis, 9 patients developed cardiac tamponade and 3 progressed to constrictive pericarditis [10,11,12,13,14,15,16,17,18,21,22]. Purulent pericarditis tends to have acute or subacute progression to constriction, and, usually, pericardiectomy is required 6 months from the onset of the disease. If constrictive pericarditis does not appear in the first 6 months, it is unexpected later on [33]. Pericardiectomy is the only ultimate treatment of pericardial constriction and should be performed when clinical symptoms are progressing despite adequate pharmacological treatment. Current studies show that total pericardiectomy carries a lower surgical mortality rate than partial or repeat surgery [34]. Purulent pericarditis is almost always fatal if untreated, and even with management has a high mortality rate [32]. A significant risk of poor prognosis is seen among patients with fever >38 °C, large pericardial effusion, cardiac tamponade, and aspirin or non-steroidal anti-inflammatory drug failure after 1 week [28]. Mortality increases with age and severe coinfections [32].

## 4. Conclusions

Though extremely uncommon, bacterial pericarditis should always be a part of differential diagnosis in a patient with progressive dyspnea, chest pain, and fever. Diagnostic evaluation of purulent pericarditis requires multidisciplinary team collaboration. Purulent pericarditis carries a high risk of mortality and complications; therefore, early recognition and targeted treatment are of the utmost importance in achieving better outcomes. Pericardiectomy appears to be the only successful strategy for the ultimate management of constrictive pericarditis.

## Data Availability

The data are not publicly available due to privacy restrictions.
